# Interaction between ERAP Alleles and HLA Class I Types Support a Role of Antigen Presentation in Hodgkin Lymphoma Development

**DOI:** 10.3390/cancers13030414

**Published:** 2021-01-22

**Authors:** Peijia Jiang, Rianne N. Veenstra, Annika Seitz, Ilja M. Nolte, Bouke G. Hepkema, Lydia Visser, Anke van den Berg, Arjan Diepstra

**Affiliations:** 1 Department of Pathology and Medical Biology, University of Groningen, University Medical Centre Groningen, 9700 RB Groningen, The Netherlands; p.jiang@umcg.nl (P.J.); r.n.veenstra@umcg.nl (R.N.V.); a.seitz@umcg.nl (A.S.); l.visser@umcg.nl (L.V.); a.van.den.berg01@umcg.nl (A.v.d.B.); 2 Department of Laboratory Medicine, Shenyang Huanggu National Defense Hospital, Shenyang 110032, China; 3 Department of Epidemiology, University of Groningen, University Medical Centre Groningen, 9700 RB Groningen, The Netherlands; i.m.nolte@umcg.nl; 4 Department of Laboratory Medicine, University of Groningen, University Medical Centre Groningen, 9700 RB Groningen, The Netherlands; b.g.hepkema@umcg.nl

**Keywords:** Hodgkin lymphoma, *ERAP1*, *ERAP2*, SNP, HLA class I, susceptibility

## Abstract

**Simple Summary:**

Hodgkin lymphoma (HL) is a common lymphoma in young adults derived from B cells. Emerging evidence suggests that antigen presentation by the malignant B cells is critically involved in HL pathogenesis. In fact, genetic variants of the antigen presenting Human Leukocyte Antigens (HLA) are strongly associated with HL susceptibility. Interestingly, the endoplasmic reticulum aminopeptidase (*ERAP*)1 and *ERAP2* genes, that code for enzymes that process antigens, also appear to be associated. In this study, we show that genetic variants of *ERAP* genes strongly affect expression levels of *ERAP1* and *ERAP2*. In addition, we find that certain *ERAP* variants interact with specific HLA class I types in HL patients. This suggests that mechanisms that determine the repertoire of antigens that are presented to the immune system, affect the chance of developing HL. Our findings therefore support a prominent role of antigen presentation in HL susceptibility.

**Abstract:**

Genetic variants in the HLA region are the strongest risk factors for developing Hodgkin lymphoma (HL), suggesting an important role for antigen presentation. This is supported by another HL-associated genomic region which contains the loci of two enzymes that process endogenous proteins to peptides to be presented by HLA class I, i.e., endoplasmic reticulum aminopeptidase 1 (*ERAP1*) and *ERAP2*. We hypothesized that *ERAP* and HLA class I type interact in HL susceptibility, as shown previously for several autoimmune diseases. We detected ERAP1 and ERAP2 expression in tumor cells and cells in the microenvironment in primary HL tissue samples. Seven *ERAP* SNPs and *ERAP1* haplotypes showed strong associations with RNA and protein levels of *ERAP1* and *ERAP2* in LCLs and HL cell lines. Analysis of HLA class I types, *ERAP* SNPs and *ERAP* haplotypes by direct genotyping or imputation from genome-wide association data in 390 HL patients revealed significant interactions between *HLA-A11*, rs27038 and the rs27038 associated *ERAP* haplotype, as well as between *HLA-Cw2* and rs26618. In conclusion, our results show that *ERAP* and HLA class I interact in genetic susceptibility to HL, providing further evidence that antigen presentation is an important process in HL susceptibility and pathogenesis.

## 1. Introduction

Hodgkin lymphoma (HL) is a heterogeneous malignancy originating from germinal center (GC) B cells. It is divided into classical HL (cHL) and nodular lymphocyte predominant HL (NLPHL), accounting for about 95% and 5% of all HL cases, respectively. In the Western world, the tumor cells are positive for Epstein-Barr virus (EBV) in around 30% of the cases [1]. Clustering of HL within families and increased prevalence in monozygotic as compared to dizygotic twins indicates a clear genetic component in the susceptibility to HL [2]. The association between specific human leukocyte antigen (HLA) alleles and the risk to develop HL further confirms presence of a genetic susceptibility component [3,4,5,6,7]. In fact, the HLA region is the strongest genetic determinant of HL susceptibility in the general population as consistently shown in several genome-wide association studies (GWAS) [8,9,10,11,12,13].

Epidemiological studies demonstrate an increased incidence of HL in patients with autoimmune diseases [14,15,16], possibly indicating a shared pathogenic mechanism. These potential associations are further strengthened by the finding of shared susceptibility loci between HL and psoriasis, ulcerative colitis and multiple sclerosis [9,12,17]. The rs27524 SNP associated with psoriasis [18], is also associated with susceptibility to HL in combined GWAS screening and validation cohorts [9]. This SNP maps within the endoplasmic reticulum amino peptidase (*ERAP*)*1* and *ERAP2* gene loci on 5q15. *ERAP1* and *ERAP2* are zinc metallopeptidases of the M1 aminopeptidase family [19]. Both enzymes trim endogenous degraded proteins to peptides to make them available for antigen presentation in the context of HLA class I [20,21,22]. These peptides need to have a specific length to effectively bind to the antigen binding groove of the HLA class I molecule [20,23,24].

The associations found for SNPs in the *ERAP* region with psoriasis [18], Behçet’s disease [25], ankylosing spondylitis [26], and Birdshot chorioretinopathy [27] are restricted to patients carrying HLA class I type Cw6, B51, B27, and A29, respectively. This indicates that *ERAP* variants influence the composition of the pool of endogenous peptides that can be presented by specific HLA class I types and as such affect disease susceptibility. *ERAP1* is highly polymorphic and multiple common isoforms exist in the general population. Functional analysis of *ERAP1* missense SNPs revealed an effect on the trimming efficiency of specific substrates [28]. In addition, multiple *ERAP1* SNPs are strong expression quantitative trait loci (eQTLs) [29,30]. *ERAP1* haplotype combinations were shown to have even more pronounced susceptibility effects [31]. *ERAP2* has only one missense SNP, rs2549782, with clear differences in trimming efficiency between the two SNP alleles [32]. This SNP is in perfect linkage disequilibrium (LD) with rs2248374, a splice-site SNP that affects *ERAP2* splicing and results in a transcript isoform with an extended exon 10 region containing a premature stop-codon. The very strong eQTL effect observed for this SNP can be explained by degradation of the *ERAP2* transcript via the nonsense mediated decay (NMD) pathway [33]. Thus, by altering ERAP1 and ERAP2 activity and/or expression, *ERAP* SNPs can have pronounced effects on the availability and repertoire of antigenic peptides available for presentation by HLA class I molecules [34].

We hypothesized that the association of rs27524 with HL susceptibility is related to differences in expression levels, trimming efficiencies and/or specificities of ERAP1 and ERAP2 protein variants and may be restricted to a subgroup of HL patients carrying specific HLA types, similar to the findings in autoimmune diseases. In this study, we first determined eQTL effects of *ERAP* SNPs and haplotypes in (HL) cell lines, EBV immortalized lymphoblastoid cells (LCLs) and HL tissue samples. We then investigated interactions between single *ERAP* SNPs and haplotypes with HLA-class I types in HL patients.

## 2. Results

### 2.1. ERAP SNP Genotyping, ERAP1 Haplotype Reconstruction and HLA Type Imputation

**Figure 1:**
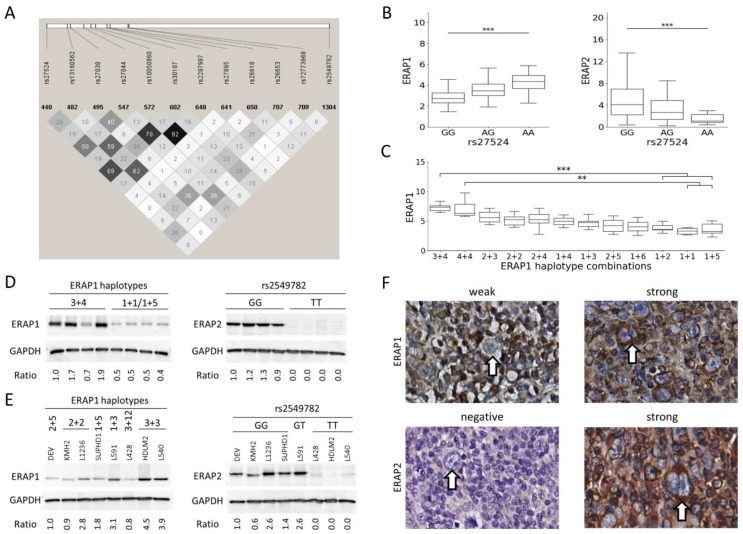
*ERAP1* and *ERAP2* expression quantitative trait loci. (**A**) Linkage disequilibrium (LD) plot of the selected SNPs in the *ERAP1* and *ERAP2* genes. The darkness of diamonds shows the strength of LD, darker colour means stronger LD, the number in the diamonds represent r^2^ between two SNPs. (**B**) eQTL analysis of rs27524 SNP in LCLs on *ERAP1* (left) and on *ERAP2* expression (right). Significance was calculated with one-way ANOVA with linear regression. (**C**) eQTL analysis of the *ERAP1* haplotype. Significance was tested using Kruskal-Wallis test with Dunn’s multiple comparison post-test. (**D**) ERAP1 and ERAP2 protein (p)QTL effect in LCLs by western blot. (**E**) pQTL analysis of the *ERAP1* haplotypes (left) and of *ERAP2* SNP rs2549782 (right) in HL cell lines by western blot. The ratio indicates the relative ERAP1 or ERAP2 protein level normalized by the GAPDH protein level. (**F**) Representative images of immunohistochemical staining of ERAP1 and ERAP2 in HL tumor tissue sections of nodular sclerosis and mixed cellularity subtype. Arrows indicate Hodgkin Reed-Sternberg cells. Statistically significant changes are indicated by ** = *p* ≤ 0.01, and *** = *p* ≤ 0.001.

*ERAP* SNP genotyping was successful for all control and HL derived LCLs, lymphoma and leukemia derived cell lines and non-GWAS HL patients. The minor allele frequencies (MAFs) were similar to those of the Utah residents with Northern and Western European ancestry (CEU) population from 1000 Genomes project (Table S1). MAFs of the typed and imputed SNPs from the GWAS HL cases were similar to each other and to the CEU frequencies (Table S1). HLA imputation of the GWAS HL cases showed an overall concordance rate of 98.2% with the PCR sequence-specific oligonucleotide probe-based HLA typing data, indicating a good imputation accuracy. These efforts resulted in genotyping data of 167 LCL and 40 lymphoma and leukemia cell lines (Table S2) for the eQTL analyses as well as high confidence HLA types and *ERAP* SNP genotype data of 390 HL patients for the interaction analyses. LD analysis for 12 selected *ERAP* SNPs showed a strong LD between several SNPs based on CEU SNP data (Figure 1A). *ERAP1* haplotype prediction showed a prediction probability of >0.9 for 374 HL patients, and these patients were included in the *ERAP1* haplotype-HLA type interaction analysis (Table S3).

### 2.2. ERAP1 and ERAP2 eQTL Analysis

EQTL analysis in LCLs showed that subjects homozygous for the risk allele A of the GWAS index SNP rs27524 had higher levels of *ERAP1* and lower levels of *ERAP2* (Figure 1B). For the 10 additional *ERAP1* SNPs, eQTL analysis revealed expression patterns similar to the index SNP, with significant differences for both *ERAP1* and *ERAP2* for most of the SNPs (Figure S1). The SNP alleles associated with higher *ERAP1* expression were associated with lower *ERAP2* expression and vice versa, consistent with the index SNP. The largest difference in *ERAP1* expression was observed between rs30187 homozygous minor as compared to major allele carriers with an effect size of 74%. The *ERAP2* SNP rs2549782 showed a very strong eQTL effect for *ERAP2*, with a lower expression level for subjects carrying the T allele (Figure S1). This *ERAP2* SNP was also significantly associated to expression levels of *ERAP1*.

*ERAP1* haplotype eQTL analyses were done for haplotype combinations that were observed in ≥5 individuals. This revealed significant differences between *ERAP1* and *ERAP2* expression levels in individuals with different haplotype combinations. Haplotype 3 and 4 heterozygous and haplotype 4 homozygous individuals were associated with high *ERAP1* expression levels. These two haplotypes both contain the risk allele of the GWAS index SNP. Haplotypes associated with a low *ERAP1* expression included haplotypes 1 and 5 that do not include the risk allele of the index SNP (Figure 1C). Haplotypes associated with high *ERAP1* expression levels showed low *ERAP2* expression levels and vice versa. (Figure 1C and Figure S2). Individuals with the *ERAP1* haplotype 3+4 combination had the highest *ERAP1* expression with a 128% higher expression level compared to individuals with a haplotype 1+5 combination.

The *ERAP2* SNP rs2549782 showed a strong eQTL effect on *ERAP2* expression in the cell line panel including 40 lymphoma and leukemia cell lines. No significant eQTL effects on *ERAP1* and *ERAP2* expression were observed for *ERAP1* SNPs and haplotypes in the cell line panel (data not shown).

### 2.3. Protein QTL Effects

Effects of *ERAP1* haplotypes and the *ERAP2* SNP genotype on protein levels were tested in eight LCLs that were selected based on a homozygous genotype for the *ERAP2* NMD associated SNP rs2549782. The *ERAP1* haplotype distribution of these eight cases included four LCLs with at least one haplotype 3 allele, and four LCLs with at least one haplotype 1 allele. In line with the results on the transcript level, haplotype 3 was associated with high ERAP1 protein and haplotype 1 with low ERAP1 protein levels. The GG genotype of rs2549782 was associated with high expression of ERAP2 and the TT genotype of rs2549782 was associated with loss of ERAP2 expression (Figure 1D). These findings are consistent with the eQTL results. Western blot analysis of HL cell lines revealed a similar pQTL pattern as observed for the LCLs (Figure 1E and Figure S3).

### 2.4. Immunohistochemistry

Immunohistochemistry was done on 10 cHL (5 nodular sclerosis and 5 mixed cellularity) cases with membranous HLA class I expression in Hodgkin Reed-Sternberg cells [35] and with homozygosity of the minor (*n* = 5) or major (*n* = 5) allele of the *ERAP2* NMD SNP. Positive ERAP1 staining was observed in Hodgkin Reed-Sternberg cells in all cases. ERAP2 was observed in both Hodgkin Reed-Sternberg cells and in cells in the microenvironment in the 5 cases that were homozygous for the minor G-allele of the *ERAP2* SNP. The 5 cases homozygous for the T-allele were consistently negative for ERAP2, both in Hodgkin Reed-Sternberg cells and cells in the microenvironment (Figure 1F). No obvious differences in ERAP1 or ERAP2 staining intensities were observed between Hodgkin Reed-Sternberg cells and reactive cells.

### 2.5. Interaction of ERAP SNPs and Haplotypes with HLA-Types in HL Patients

**Figure 2:**
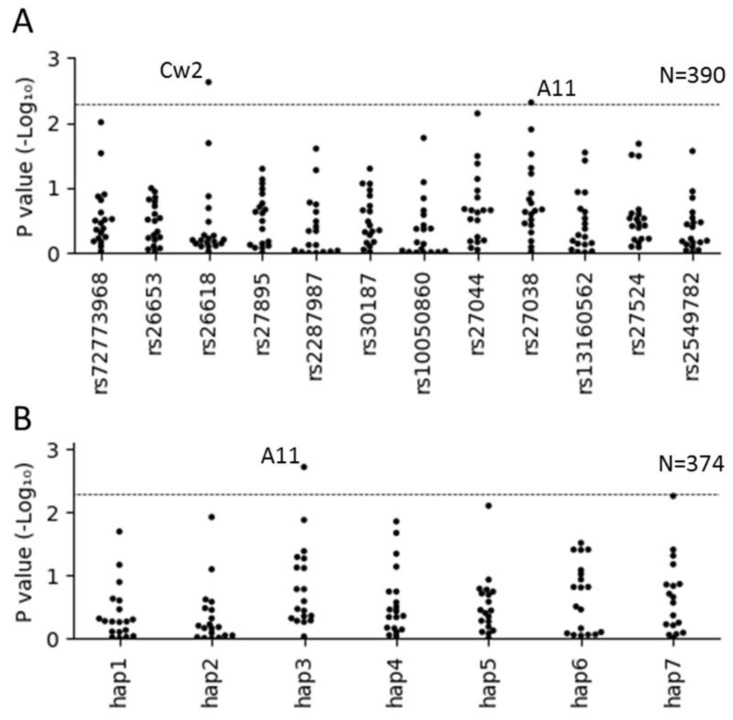
Association analysis of *ERAP1* and *ERAP2* SNPs and haplotypes with HLA phenotype in HL patients. (**A**) SNPs are ordered by chromosomal position. Genotyped or best guess *ERAP* genotypes and typed or best guess HLA phenotypes were used. (**B**) *ERAP1* haplotypes are ordered from high to low frequency in the CEU population. The best guess *ERAP1* haplotypes and typed or best guess HLA phenotypes were used. Samples were excluded if the maximum probability of a haplotype combination was <0.9. Logistic regression analysis was used to determine significance of associations. A *p*-value of <0.005 (dashed line) was considered significant.

The *ERAP* SNP dosage—HLA phenotype interaction analysis revealed a significant association for rs27038 with *HLA-A11* and for rs26618 with *HLA-Cw2* (Figure 2A). These associations remained significant in the sensitivity analyses (Figure S4). For *ERAP1* haplotypes, we identified a significant interaction for *ERAP1* haplotype 3 with *HLA-A11* (Figure 2B). This association also remained significant in the sensitivity analyses. Results of the initial and sensitivity analyses are summarized in Tables S4 and S5. We did not find an association between the rs27524 SNP and *HLA-Cw6* in HL patients, in contrast to previous results in psoriasis [18]. EBV stratified analyses did not reveal any significant associations.

The *ERAP* SNP genotype—HLA genotype analysis revealed interactions for rs26618 with *HLA-Cw2*, and for rs27038 with *HLA-A11* and *HLA-A68*. These interactions remained significant in the sensitivity analyses (Figure S5). For *ERAP1* haplotypes, four significant interactions with HLA genotypes were identified, i.e., *ERAP1* haplotype 3 with *HLA-A11* and *HLA-B35*, haplotype 4 with HLA-Cw7 and haplotype 7 with *HLA-Cw2* (Figure S5). Results of the initial and sensitivity analysis are summarized in Tables S4 and S5. Again, no significant associations were found in EBV+ and EBV− subgroups.

## 3. Discussion

In this study we explored the relevance of the previously reported association of an *ERAP* SNP with HL susceptibility in the context of specific HLA types. We showed strong eQTL effects of *ERAP* SNPs and haplotypes consistent with previous studies [30,36]. The risk allele (A allele) of the GWAS index SNP rs27524 and haplotypes 3 and 4 carrying this risk allele were associated with high *ERAP1* and low *ERAP2* expression. The effect of *ERAP1* SNPs and haplotypes on *ERAP2* and vice versa can be attributed to the strong LD of variants in this genomic region. With regards to HL susceptibility, significant interactions were observed between *ERAP1* SNP rs27038 and *HLA-A11*, *ERAP1* SNP rs26618 and *HLA-Cw2*, and *ERAP1* haplotype 3 and *HLA-A11*. This suggests that mechanisms that determine the repertoire of antigens that are presented by HL precursor cells, affect the chance of developing full-blown HL.

Proper tumor-specific antigen presentation is an essential process in the induction of a functional anti-tumor immune response and requires expression of HLA class I and ERAP proteins. In many cancer types, tumor cells attempt to escape from immune responses by abolishing cell surface HLA class I expression [37]. *ERAP1* and *ERAP2* expression is also frequently altered in melanoma, acute myeloid leukemia, gastric adenocarcinoma, HPV-induced malignancies and renal clear cell carcinoma, as another immune escape mechanism [38,39,40,41]. In HL, expression of membranous HLA class I by tumor cells is retained in a significant proportion of cases [35,42,43]. The differences we observed in ERAP expression patterns in HLA class I positive HL cases were associated with the genotype of the subjects and there was no down regulation of ERAP expression in the tumor cells relative to normal cells in the tumor microenvironment. Thus, the tumor cells of HLA class I positive HL cases show normal ERAP1 and ERAP2 expression patterns. This means that, in combination with the previously reported expression of other antigen processing proteins such as TAP1 and TAP2, the essential components of the antigen presentation pathway are present in the tumor cells of HL [44].

The function of ERAP molecules has not been studied in HL, but in other diseases and model systems ERAP is strongly linked to antigen presentation and immune responses. Deficiency in ERAP1 expression was shown to result in unstable and highly immunogenic peptides presented in the context of HLA class I [45]. Indeed, altered ERAP1 expression influences the effectiveness of cytotoxic CD8+ T-cell anti-tumor immune responses in the context of solid cancer, melanoma and a T-cell lymphoma mouse model [40,46,47]. Another study showed that lack of ERAP2 expression decreased NK cell activation in choriocarcinoma cell lines [48]. In addition, differential substrate specificity based on combinations of *ERAP* missense SNPs affects the peptidome, which may reduce recognition of tumor cells by CD8+ T cells and NK cells [47,49]. For example, *ERAP1* missense SNP alleles rs30187-T and rs27044-C affect peptide specificity, alter the peptide pool available for being presented in the context of HLA class I, and potentially facilitate immune evasion [28,50]. Besides the direct effects of missense SNPs on expression levels and substrate specificity, the ratio between ERAP1 and ERAP2 may also influence peptide trimming efficiency due to their functional coordination by forming a heterodimer [51]. We showed that the A allele of the rs27524 HL associated risk SNP results in high ERAP1 and low ERAP2 expression, which affects peptide trimming efficiency and is expected to associate with a restricted range of peptides in the peptidome. This may result in a skewed anti-tumor CD8+ T cell response and contribute to escape of HL tumor cells from effective anti-tumor immune surveillance. This concept fits well with ERAP-dependent mechanisms as proposed for multiple other cancer types [40,46,47].

We demonstrated a significant interaction between the C allele of rs26618 and *HLA-Cw2* in HL patients. Rs26618 is a missense SNP that is expected to define peptide specificity and the C allele is associated with high ERAP2 expression. We also identified interactions of the rs27038 A allele and the associated haplotype 3 with *HLA-A11. HLA-A11* was previously reported to be associated with HL [52], although this association was not confirmed in later studies [53,54]. This HLA type can present peptides from EBV and induce cytotoxic T cell responses [55,56]. Both the intronic rs27038 A allele and the *ERAP1* haplotype 3 are associated with high ERAP1 and low ERAP2 expression. Thus, this combination of alleles might have an impact on the presentation of tumor-associated antigenic peptides by HLA-A11. Unfortunately, due to a relatively small group size of EBV+ HL patients, we could not determine whether there was an EBV specific effect and a potential relation should be further explored in a larger EBV+ HL cohort. Regarding HL subtype, we consider the interactions to reflect cHL, as these interactions remained significant in the sensitivity analyses that excluded NLPHL cases.

In previous studies on autoimmune diseases, interactions of *ERAP* were shown with known HLA susceptibility types, i.e., rs27524 with *HLA-Cw6* in psoriasis [18], rs17482078 with *HLA-B51* in Behçet’s disease [25], rs30187 with *HLA-B27/HLA-B40* in ankylosing spondylitis [26], and rs7705093 with *HLA-A29* in birdshot chorioretinopathy [27]. In contrast, the HLA types identified in this HLA-*ERAP* interaction study, i.e., *HLA-A11* and *HLA-Cw2*, have not been associated with HL susceptibility [6,7]. We also did not find *ERAP*-HLA interactions with HLA types that have previously been associated with HL susceptibility. This might be caused by less strong associations between *ERAP* and HLA types in HL and limited power due to low frequencies of certain HLA risk types in our HL cohort. Nonetheless, the interactions observed in the current study suggest that *ERAP* missense SNPs have an effect on the peptide pool available for being presented by specific HLA types and as such influence susceptibility to HL.

## 4. Materials and Methods

### 4.1. Patient and Control Samples

Patients included in this study were retrieved from previously published HLA typing (*n* = 332) and GWAS (*n* = 304) studies, with an overlap of 278 HL patients [6,11]. Most patients were diagnosed with HL in multiple centers in the Northern region of the Netherlands between 1987–2010. In addition, 56 new HL patients were included who were diagnosed between 2010–2012 at the University Medical Centre Groningen, the Netherlands (Figure S6). There were 397 cHL and 17 NLPHL cases, according to the World Health Organization classification system [57]. EBV status was determined previously by EBER in situ hybridization (ISH) [6,9]. Additionally, 97 healthy controls were included in this study. This research has been approved by the Medical Ethical Committee of the University Medical Centre Groningen on 10 November 2011 (ethic code: METc 2004/219) and was conducted in accordance with the Declaration of Helsinki. Informed consent was given by all patients.

### 4.2. LCL Generation and Cell Line Culture

LCLs were generated by infection of peripheral blood mononuclear cells (PBMCs) from 97 anonymized healthy donors and 70 successfully treated HL patients with B95.8 EBV virus using standard procedures. Forty-four of these HL patients were also included in the HLA typing study and 26 were from the new HL cases. LCLs were cultured in RPMI 1640 (Lonza BioWhittaker, Walkersville, MD, USA) supplemented with 10% Fetal Bovine Serum (FBS, HyClone Thermo Scientific, Waltham, MA, USA) and 100U/mL of Penicillin/Streptomycin and 1% Ultraglutamine (BioWhittaker, Basel, Switzerland). All HL cell lines except DEV were obtained from DSMZ (German Collection of Microorganisms and Cell Cultures GmbH, Braunschweig, Germany). The DEV cell line was made in our group [58]. These cell lines were cultured in RPMI 1640 with 20% FBS (DEV, L540 and HDLM2), 10% (KMH2, L591 and L1236) or 5% (L428) FBS and Penicillin/Streptomycin and Ultraglutamine. SUPHD1 was cultured in McCoys5A with 20% FBS and Penicillin/Streptomycin and Ultraglutamine. Thirty-two additional lymphoma and leukemia cell lines were used to extend the cell line panel (Table S2). These cell lines were cultured as described previously [59,60]. Mycoplasma tests were done at a regular basis and cell line identities were confirmed through short tandem repeat (STR) analysis.

### 4.3. ERAP SNP Selection, Genotyping, Imputation and Haplotype Reconstruction

We selected all *ERAP1* and *ERAP2* missense SNPs with a minor allele frequency (MAF) above 5% based on public data from CEU cohort of 1000 Genomes phase 3 (ftp://ftp.1000genomes.ebi.ac.uk/vol1/ftp/release/20130502). This resulted in 10 SNPs, 9 mapping to the *ERAP1* locus and one mapping to the *ERAP2* locus. In addition, we included (1) the index rs27524 SNP identified in our previous GWAS study [9], and (2) rs27038 and rs13160562 SNPs showing a strong eQTL effect according to the GTEx portal (https://www.gtexportal.org/home/) and a recent study by Harson et al. [30]. LD analysis for all of the 13 above mentioned SNPs in the CEU cohort of the 1000 Genomes phase 3 using Haploview v4.2 [61] showed perfect LD between rs2287987 and rs17482078. So only rs2287987 was selected for genotyping. This resulted in a final selection of 11 SNPs mapping at the *ERAP1* locus and one *ERAP2* SNP.

For 304 HL cases, SNP data were extracted from our previous GWAS data by PLINK v1.07 (http://pngu.mgh.harvard.edu/purcell/plink/) [62] (Figure S6). Six (rs27524, rs13160562, rs30187, rs10050860, rs26618, rs2549782) of the 12 SNPs selected for analysis were genotyped within the GWAS. The remaining six SNPs were imputed using the HRC reference panel (Version r1.1 2016) on the Michigan Imputation Server (https://imputationserver.sph.umich.edu/) [63]. The average probability was >99% for each of the SNPs. The genotype with the highest probability was used for the analyses (i.e., best guess genotype). Eight of the 304 × 6 imputed genotypes with an imputation quality score r^2^ < 0.95 were excluded in the sensitivity analyses.

For the 97 healthy controls, 110 HL patients not included in the GWAS and for the 40 cell lines, genomic DNA was isolated following routine protocols. SNP genotyping was done in triplicate on the Taqman 7900HT fast real-time PCR system using Taqman assays (Thermo Fisher Scientific, Waltham, MA, USA) (Table S1) in a final volume of 5 μL using 5 to 10 ng DNA in a 384-well plate (Applied Biosystems, Waltham, MA, USA). Genotypes were called by QuantStudio™ Real-Time PCR Software. As a quality check, frequencies of the SNPs were compared to those of the CEU population from the 1000 Genomes Project [64].

*ERAP1* haplotype reconstructions were carried out using genotype data of the 11 selected *ERAP1* SNPs using PHASE v2.1.1 [65,66]. The haplotype combination with the highest probability (i.e., best guess haplotype) was assigned. Samples with haplotype combinations with predicted probabilities of <0.9 were excluded (*n* = 16) from the interaction analyses.

### 4.4. HLA Typing and Imputation

For 332 HL cases, HLA types were determined using a sequence-specific oligonucleotide PCR method as described in our previous study [6]. For 32 of the new HL cases, HLA typing was performed following the same approach. For 26 HL patients (including 9 cHL and 17 NLPHL) from the GWAS and for 12 HL patients for whom HLA typing failed for some of the HLA class I alleles, HLA types were defined using the SNP-based HLA imputation R-package HIBAG [67]. Data not meeting the imputation quality thresholds were set to missing. Eight poorly imputed HLA types with a prediction probability <0.8 were excluded in the indicated sensitivity analyses. HLA imputation was done for all HL cases included in the GWAS. The imputation quality was assessed for the 278 patients that were included both in the GWAS and the HLA typing cohort by determining the concordance between real and imputed HLA types.

### 4.5. qRT-PCR

Total RNA was isolated from LCL and lymphoma cell lines using TRIzol^®^ reagent (Thermo Fisher Scientific, Waltham, MA, USA) following the protocol of the manufacturer. Concentration of RNA samples was measured on the Nanodrop-1000 spectrophotometer and quality was checked on a 1% agarose gel. cDNA was synthesized from 500 ng total RNA in 20 μL reaction volume using Superscript II Reverse Transcriptase Kit and Random primers in accordance with the protocol of the manufacturer (Thermo Fisher Scientific, Waltham, MA, USA). The qPCR was performed in a total volume of 10 μL with 300 nM primers, 1 ng cDNA and 5 μL SYBR^®^ Green Real-Time PCR Master Mixes (Thermo Fisher Scientific Inc., Waltham, MA, USA) on the Lightcycler 480 (Roche, Penzberg, Germany), and in triplicate for each sample. Relative expression levels were determined using 2-ΔCp using TATA-Box Binding Protein (TBP) as a housekeeping gene. Primer sequences used for amplification of *ERAP1*, *ERAP2* and *TBP* are listed in Table S6.

### 4.6. Western Blot

Cells (about 1–20 × 10^6^) of eight LCLs that were selected based on homozygosity of the *ERAP2* SNP and cells of the eight Hodgkin lymphoma cell lines were lysed in cell lysis buffer (#9803, Cell Signaling Technology, Danvers, MA, USA) with 1 mM phenylmethanesulphonyl fluoride (PMSF) on ice for 30–45 min. The protein concentration was measured using the Pierce™ BCA Protein Assay Kit (#23227, Thermo Fisher Scientific Inc., Waltham, MA, USA). Twenty μg of protein was electrophoresed on a 6% SDS-PAGE gel and transferred to a nitrocellulose membrane using standard protocols. Blots were blocked in TBST + 5% ELK milk powder for 60 min and incubated with primary antibodies (Goat anti-human ERAP1 antibody 1:500, AF2334, R&D Systems, Minneapolis, USA; and Goat anti-human ERAP2 antibody, 1:1000, AF3830, R&D Systems) overnight at 4 °C. After washing, blots were incubated with HRP-conjugated rabbit anti-goat antibody (#P0449, Dako, Glostrup, Denmark, 1:1000 dilution), which was followed by an incubation with HRP-conjugated goat anti-rabbit antibody (#P0448, Dako, Glostrup, Denmark, 1:1000 dilution). SuperSignal WestPico Chemiluminescent Substrate (#34078, Thermo Fisher Scientific Inc., Waltham, MA, USA) was used to visualize ERAP1 and ERAP2 protein. Glyceraldehyde-3-Phosphate Dehydrogenase (GAPDH) was used as a housekeeping gene.

### 4.7. Immunohistochemistry

Primary formalin-fixed paraffin-embedded (FFPE) tissue sections of 10 cHL cases (5 nodular sclerosis, 5 mixed cellularity) selected based on membranous expression of HLA class I and *ERAP2* SNP genotype (5 homozygous minor and 5 homozygous major allele of the index SNP) were deparaffinized using xylene and ethanol and subjected to heat-induced antigen retrieval in 1mM EDTA pH = 8.0 for 15 min. Endogenous peroxidase was blocked using 0.3% H2O2 for 30 min and avidin and biotin were blocked using the avidin/biotin blocking kit (# SP-2001, Vector Laboratories, Burlingame, CA, USA). Goat anti-human ERAP1 antibody (AF2334, R&D Systems, Minneapolis, MN, USA) at 1:50 dilution and Goat anti-human ERAP2 antibody (AF3830, R&D Systems, Minneapolis, MN, USA) at 1:100 dilution in PBS + 1% BSA were incubated at room temperature for 60 min. After washing, slides were incubated with biotin-conjugated rabbit anti-goat antibody (#6165-08, Southern Biotech, Birmingham, AL, USA) at 1:100 dilution for 30 min, followed by an incubation with streptavidin-HRP (#P0397, Dako, Glostrup, Denmark) at 1:300 dilution at room temperature for 30 min. 3,3′-Diaminobenzidine (DAB) staining was used to visualize the protein and slides were counterstained with hematoxylin. Scoring was performed in an unbiased way, without knowing the *ERAP1* and *ERAP2* genotypes. We scored the staining intensity for ERAP1 and ERAP2 based on the pattern in the majority of the Hodgkin Reed-Sternberg cells as negative, weak, intermediate or strong. For both ERAP1 and ERAP2, staining patterns were evaluated separately for Hodgkin Reed-Sternberg cells and cells in the microenvironment.

### 4.8. Statistical Analysis

One-way analysis of variance (ANOVA) with a post-hoc test for linear trend for number of alleles and comparison between the mean of each genotype was employed to test for eQTL effects of *ERAP1* and *ERAP2* SNP genotypes. A Kruskal-Wallis test with a Dunns post-hoc test was applied to analyze the eQTL effect of *ERAP1* haplotypes. Analyses were performed using GraphPad Prism 5.0 (GraphPad Software, Inc., San Diego, CA, USA) and python3.7 with packages of SciPy (https://www.scipy.org/).

Association analysis testing for an interaction effect of *ERAP* (additive model) and HLA phenotypes were performed in PLINK v1.07 using logistic regression by comparing mean number of risk alleles of *ERAP* SNP allele or *ERAP1* haplotype between HLA type carriers and non-carriers. In addition, *ERAP*—HLA genotype interactions were determined by comparing observed with expected frequencies using Chi-square tests. *ERAP1* haplotype analyses were performed for individuals for whom the probability of the haplotype reconstruction was >0.9 using PHASE. We tested all of the 18 HLA types with an allele frequency >5% in the study population. Power calculations using the online Clincalc tool (https://clincalc.com/stats/Power.aspx) revealed a power of 80% to detect a relative risk ratio of interaction of 1.28 and more. Considering multiple testing correction and LD in both the HLA and the *ERAP* gene regions we regarded a *p*-value < 0.005 as significant.

*ERAP* allele dosage/HLA phenotype association analyses were done on the entire group of 390 HL cases using best guess genotypes for all *ERAP* SNP and HLA imputation results. Since imputation results can be imprecise, we performed a sensitivity analysis by setting imputed SNP genotypes and imputed HLA types with an imputation accuracy (r^2^) of <0.95 and <0.8, respectively, to missing (*n* = 390). Another sensitivity analysis was done only including cases with direct HLA typing data (*n* = 364). This resulted in exclusion of 9 cHL and all 17 NLPHL cases as they only had imputed HLA types. In addition, we performed *ERAP* genotype/HLA genotype association analyses using the same sensitivity groups as described above. Besides analyzing the total HL group, we also explored associations in the EBV positive and negative subgroups (only using best guess *ERAP* genotypes and HLA-types of all 390 cases without filtering for imputation quality).

## 5. Conclusions

In conclusion, our data indicate a role of *ERAP1* and *ERAP2* variants in the development of HL in the context of specific HLA types. Given the importance of ERAP1 and ERAP2 in trimming antigenic peptides and the known and very strong associations of the HLA region with HL susceptibility, our data support an important role of antigen presentation in HL susceptibility and pathogenesis.

## Data Availability

SNP genotype data from CEU cohort of 1000 Genomes phase 3 are publicly available (ftp://ftp.1000genomes.ebi.ac.uk/vol1/ftp/release/20130502).

## Supplementary Materials

The following are available online at https://www.mdpi.com/2072-6694/13/3/414/s1, Figure S1. Overview of eQTL effects of all 12 missense SNPs in *ERAP1* and *ERAP2*. Figure S2. eQTL analysis of *ERAP1* haplotype on *ERAP2* expression in lymphoblastoid cell lines. Figure S3. Western blot of ERAP1 and ERAP2 protein. Figure S4. Sensitivity analyses for testing the association between genotypes of 12 missense SNPs of the *ERAP1* and *ERAP2* gene loci and HLA-phenotype in HL patients. Figure S5. Sensitivity analyses for testing the association between 12 missense SNPs genotypes of the *ERAP1* and *ERAP2* gene loci and HLA-genotype HL. Figure S6. Schematic representation of the HL cases included in this study. Table S1. Minor allele frequencies of selected SNPs in the *ERAP1* and *ERAP2* genes in LCL controls and HL patients. Table S2. Cell lines used in this study. Table S3. *ERAP1* haplotypes in CEU controls and HL patients. Table S4. *ERAP* SNP—HLA allele interactions with (nearly) significant associations. Table S5. *ERAP1* haplotype—HLA type interactions with (nearly) significant associations. Table S6. Sequence of the primers used for qRT-PCR.
