# Supplementary material for: Interaction between ERAP Alleles and HLA Class I Types Support a Role of Antigen Presentation in Hodgkin Lymphoma Development
Source: Cancers (Basel). 2021 Jan 22;13(3):414. doi: 10.3390/cancers13030414 (PMC7865538; doi:10.3390/cancers13030414)
Supplement: Supplementary file 1 [file cancers-13-00414-s001.pdf]

cancers

Supplementary materials

Interaction between ERAP Alleles and HLA Class I Types Support a Role of Antigen Presentation in Hodgkin Lymphoma Development

Peijia Jiang [1,2], Rianne N. Veenstra [1], Annika Seitz [1], Ilja M. Nolte [3], Bouke G. Hepkema [4], Lydia Visser [1], Anke van den Berg [1] and Arjan Diepstra [1,*]

1 Department of Pathology and Medical Biology, University of Groningen, University Medical Centre Groningen, 9700 RB Groningen, The Netherlands; p.jiang@umcg.nl (P.J.); r.n.veenstra@umcg.nl (R.N.V.); a.seitz@umcg.nl (A.S.); l.visser@umcg.nl (L.V.); a.van.den.berg01@umcg.nl (A.v.d.B.)
2 Department of Laboratory Medicine, Shenyang Huanggu National Defense Hospital, Shenyang 110032 , China
3 Department of Epidemiology, University of Groningen, University Medical Centre Groningen, 9700 RB Groningen, The Netherlands; i.m.nolte@umcg.nl
4 Department of Laboratory Medicine, University of Groningen, University Medical Centre Groningen, 9700 RB Groningen, The Netherlands; b.g.hepkema@umcg.nl

* Correspondence: a.diepstra@umcg.nl

Citation: Jiang, P.; Veenstra, R.N.; Seitz, A.; Nolte, I.M.; Hepkema, B.G.; Visser, L.; van den Berg, A.; Diepstra, A. Interaction between ERAP Alleles and HLA Class I Types Support a Role of Antigen Presentation in Hodgkin Lymphoma Development. *Cancers* **2021**, *13*, 414. https://doi.org/10.3390/cancers13030414

Received: 8 January 2021
Accepted: 19 January 2021
Published: 22 January 2021

Publisher's Note: MDPI stays neutral with regard to jurisdictional claims in published maps and institutional affiliations.

Copyright: © 2021 by the authors. Licensee MDPI, Basel, Switzerland. This article is an open access article distributed under the terms and conditions of the Creative Commons Attribution (CC BY) license (http://creativecommons.org/licenses/by/4.0/).

Supplementary Materials

Table S1. Minor allele frequencies of selected SNPs in the *ERAP1* and *ERAP2* genes in LCL controls and HL patients.

SNP	Major allele	Minor allele	Gene	Description	SNP genotyping assay ID[1]	Minor allele frequency			
						CEU[2] (n=99)	genotyped controls (n=97)	genotyped patients (n=110)	GWAS patients (n=304)
**rs27524**	**G**	**A**	***ERAP1***	**intron (GWAS index)**	C_3056837_10	0.33	0.37	0.43	0.39
rs13160562	G	A	*ERAP1*	intron (top eQTL)	C_3056855_10	0.36	0.3	0.28	0.27
rs27038	G	A	*ERAP1*	intron (top eQTL)	C_794764_20	0.15	0.14	0.21	0.16[3]
rs27044	C	G	*ERAP1*	Q730E	C_3056870_10	0.26	0.28	0.31	0.32[3]
rs10050860	C	T	*ERAP1*	D575N	C_3056876_10	0.28	0.24	0.19	0.18
rs30187	C	T	*ERAP1*	K528R	C_3056885_10	0.31	0.3	0.36	0.36
rs2287987	T	C	*ERAP1*	M349V	C_3056893_20	0.26	0.24	0.19	0.18[3]
rs27895	C	T	*ERAP1*	G346D	C_794792_10	0.08	0.06	0.07	0.07[3]
rs26618	T	C	*ERAP1*	I276M	C_3056894_10	0.23	0.29	0.25	0.27
rs26653	G	C	*ERAP1*	R127P	C_794818_30	0.28	0.22	0.32	0.27[3]
rs72773968	G	A	*ERAP1*	T12I	C_98862195_10	0.12	0.14	0.09	0.16[3]
rs2549782	T	G	*ERAP2*	N392K	C_3282749_20	0.50	0.47	0.50	0.47

[1] ID from Thermo Fisher Scientific; [2] 1000G population of 99 Utah residents with Northern and Western European ancestry from the CEPH collection; [3] Minor allele frequencies of imputed SNPs.

Table S2. Cell lines used in this study.

Cell line	Disease
OCIAML3	AML
NB4	AML
THP1	AML
KARPAS422	AML
MOLM13	AML
HL60	AML
IMSM2	AML
BL65	BL
ST486	BL
CA46	BL
Raji	BL
DG75	BL
Ramos	BL
JVM3	CLL
MO1043	CLL
U2932	DLBCL
SUDHL10	DLBCL
OCILY3	DLBCL
SUDHL2	DLBCL
SUDHL5	DLBCL
SUDHL6	DLBCL
WSUDLCL2	DLBCL
WSUFSCCL	DLBCL
SUDHL4	DLBCL
SC-1	FL
Dohh2	FL
DEV	NLPHL
HDLM2	cHL

KMH2	cHL
L1236	cHL
L540	cHL
SUPHD1	cHL
L428	cHL
L591	cHL
Granta519	MCL
K1106P	PMBL
HUT78	TCL
Jurkat	TCL
Karpas299	TCL
SR786	TCL

AML, Acute myeloid leukaemia; BL, Burkitt's lymphoma; CLL, Chronic lymphocytic leukaemia; DLBCL, Diffuse large B-cell lymphoma; FL, follicular lymphoma; NLPHL, nodular lymphocyte predominant Hodgkin lymphoma; cHL, classical Hodgkin lymphoma; MCL, Mantle cell lymphoma; PMBL, primary mediastinal B cell lymphoma; TCL, T cell lymphoma.

Table S3. *ERAP1* haplotypes in CEU controls and HL patients.

**SNP**	***ERAP1* haplotype**															
	**1**	**2**	**3**	**4**	**5**	**6**	**7**	**8**	**9**	**10**	**11**	**12**	**13**	**14**	**15**	**others**
**rs27524**	G	G	A	A	G	G	A	A	G	G	G	A	G	G	A	
**rs13160562**	A	G	G	G	A	G	G	G	G	G	A	G	A	A	G	
**rs27038**	G	G	A	G	G	G	G	G	A	G	G	A	G	G	G	
**rs27044**	C	C	G	G	C	C	C	C	G	C	C	C	G	C	C	
**rs10050860**	T	C	C	C	C	C	C	C	C	C	C	C	C	T	T	
**rs30187**	C	C	T	T	C	C	T	C	T	C	C	C	T	C	T	
**rs2287987**	C	T	T	T	T	T	T	T	T	T	T	T	T	T	T	
**rs27895**	C	C	C	C	C	T	C	C	C	C	C	C	C	C	C	
**rs26618**	T	C	T	T	T	T	T	C	T	T	C	T	T	T	T	
**rs26653**	G	G	C	G	G	C	C	G	C	G	G	G	C	G	C	
**rs72773968**	G	G	G	A	G	G	G	G	G	G	G	G	G	G	G	
**Haplotype frequencies**																
**CEU (%)[1]**	26	17	12	12	8.6	7.6	5.1	3.5	2.0	2.0	1.5	1.0	1.0	0.5	0.5	0.0
**LCL controls (%)**	21	25	10	14	8.3	6.3	4.7	3.1	0.5	1.6	0.0	0.0	0.5	0.0	0.0	4.7
**HL cases (%)[2]**	18	23	15	14	9.0	6.5	4.9	4.4	0.8	2.2	0.0	0.0	0.1	0.1	0.0	2.6

[1] CEU, Utah residents with Northern and Western European ancestry from the CEPH collection available in the 1000 Genomes Project (n = 99). [2] All HL patients included in interaction analyses regardless of imputation quality (n = 374).

Table S4. *ERAP* SNP - HLA allele interactions with (nearly) significant associations.

**Patient group \ *ERAP* SNP - HLA combination**	**rs26618-Cw2**	**rs27038-A11**	**rs27044-B35**	**rs27038-A68**	**rs10050860-B8**	**rs2287987-B8**
**HLA phenotype**						
Typed or best-guess SNP genotype and typed or best guess HLA (n=390)	**0.0024**	**0.0049**	0.0072	0.0127	0.0171	0.0250
Typed or best guess SNP genotype (r^2>0.95) and typed or best guess HLA (r^2>0.8) (n=390)	**0.0013**	**0.0041**	0.0128	0.0109	0.0108	0.0162
Typed or best guess SNP genotype (r^2>0.95) and typed HLA (n=364)[1]	**0.0012**	**0.0034**	0.0332	0.0177	**0.0035**	0.0056
**HLA genotype**						
Typed or best-guess SNP genotype and typed or best guess HLA (n=390)	**0.0031**	**0.0021**	**0.0046**	**0.0046**	0.0098	0.0141
Typed or best guess SNP genotype (r^2>0.95) and typed or best guess HLA (r^2>0.8) (n=390)	**0.0024**	**0.0016**	0.0062	**0.0024**	**0.0030**	**0.0046**
Typed or best guess SNP genotype (r^2>0.95) and typed HLA (n=364)[1]	**0.0020**	**0.0015**	0.0139	**0.0033**	**0.0020**	**0.0031**

[1] In this group all NLPHL cases (n=17) and a few cHL cases (n=9) are excluded.P-values marked bold are < 0.005 and considered significant.

Table S5. *ERAP1* haplotype - HLA type interactions with (nearly) significant associations.

Patient group \ *ERAP1* haplotype - HLA combination	hap3 – A11	hap3 – B35	hap4 – Cw7	hap7 – Cw5
ERAP1 haplotype and HLA phenotype (n=374)	**0.0019**	0.0132	0.0139	0.0055
ERAP1 haplotype and HLA genotype (n=374)	**0.0006**	**0.0023**	**0.0047**	**0.0005**

P-values marked in bold are < 0.005 and considered significant. Sixteen patients with *ERAP1* haplotype prediction probability < 0.9 were excluded from this interaction analysis.

Table S6. Sequence of the primers used for qRT-PCR.

Gene	Forward primer (5'--> 3')	Reverse primer (5'--> 3')
ERAP1	CCGTATCCCCTACCCAAACAA	AGTTTTCCATAGCACCAGACTGAA
ERAP2	AGGTGATGGCTTTGAAGGGTT	TGCCTGGGTTGGCTCAAAAT
TBP	GCCCGAAACGCCGAATAT	CCGTGGTTCGTGGCTCTCT

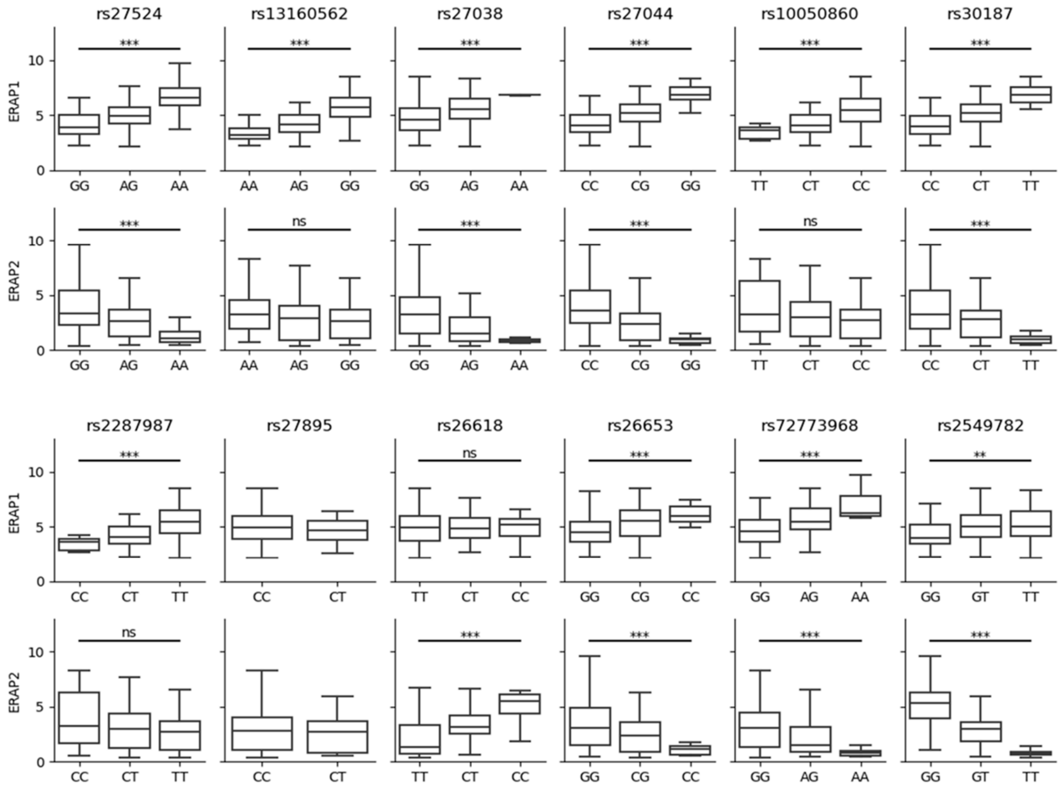

Figure S1. Overview of eQTL effects of all 12 missense SNPs in *ERAP1* and *ERAP2*. One-way ANOVA with a linear trend for the number of minor alleles with mean expression of each genotype group was used to establish significance. *: $p < 0.05$, **: $p < 0.01$, ***: $p < 0.001$.

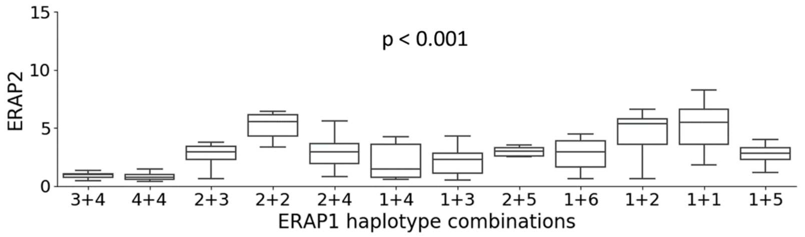

Figure S2. eQTL analysis of the effect of *ERAP1* haplotype on *ERAP2* expression in LCLs. For significance testing the Kruskal-Wallis test was used.

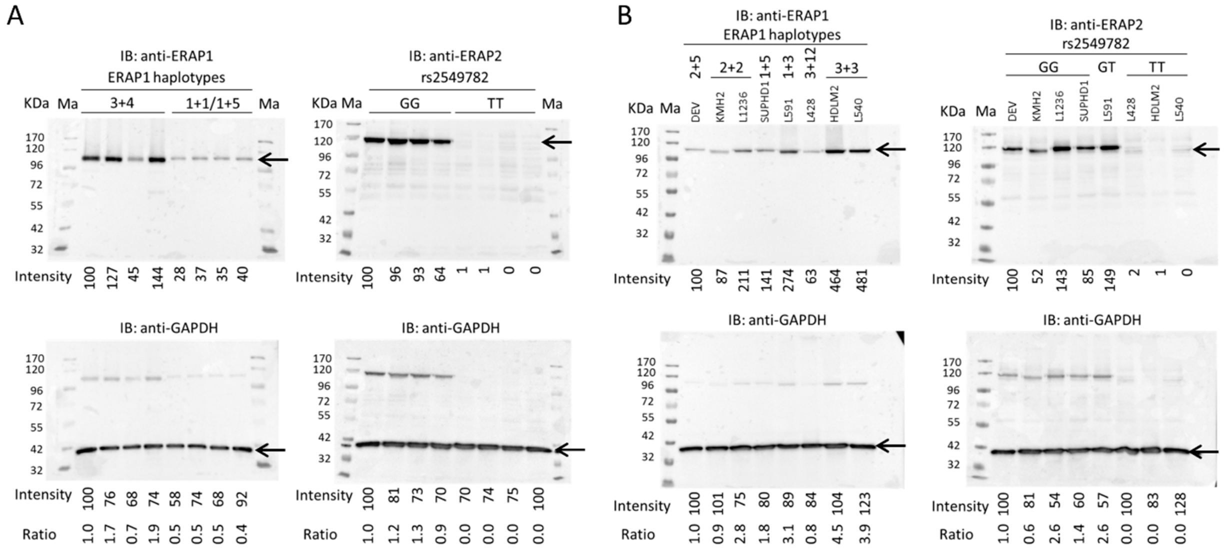

Figure S3. ERAP1 and ERAP2 western blots. (**A**) ERAP1 and ERAP2 protein expression based on ERAP1 haplotype combinations (left) and ERAP2 SNP (right) in LCLs. (**B**) ERAP1 and ERAP2 protein expression based on ERAP1 haplotype combinations (left) and ERAP2 SNP (right) in HL cell lines. Position of target protein was indicated by arrow. Intensity of target protein bands was quantified using Image lab 6.0. The blot membranes were first incubated with ERAP1 or ERAP2 antibody. After signal capture, the ERAP1 or ERAP2 antibody was stripped from the blot and the same blot was used for detection of GAPDH. Ma, molecular weight marker.

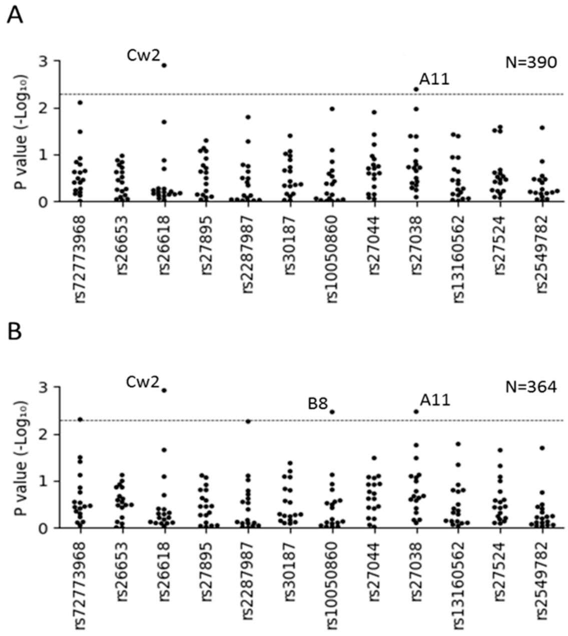

Figure S4. Sensitivity analyses for testing the association between genotypes of 12 missense SNPs of the *ERAP1* and *ERAP2* gene loci and HLA-phenotype in HL patients. (**A**) The association analyses between genotyped or best guess ($r^2 > 0.95$) *ERAP* SNP alleles and typed or best guess ($r^2 >$ 0.8) HLA phenotype (n=390). (**B**) The association analyses between genotyped or best guess ($r^2 >$ 0.95) *ERAP* SNP alleles and typed HLA phenotype (n=364). Logistic regression analysis was used to determine significance of the associations. A p-value < 0.005 (dashed line) was considered significant.

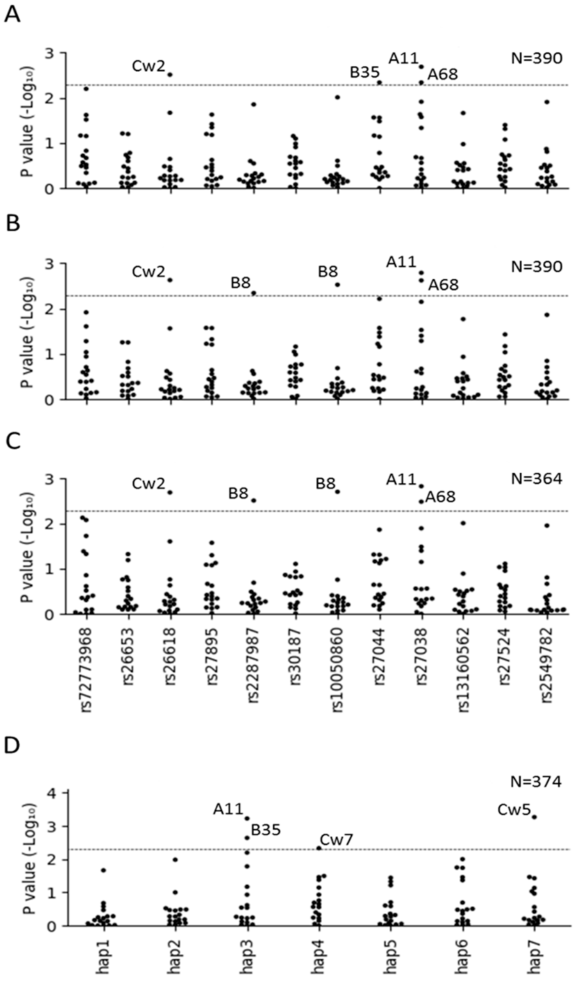

Figure S5. Sensitivity analyses for testing the association between 12 missense SNP genotypes of the *ERAP1* and *ERAP2* gene loci and HLA-genotype in HL. (**A**) The association analyses between genotyped or best guess imputed *ERAP* SNP and typed or best guess imputed HLA genotype (n=390), regardless of imputation quality. (**B**) Association analyses between genotyped or best guess ($r^2 > 0.95$) *ERAP* SNP and typed or best guess ($r^2 > 0.8$) HLA genotype (n=390). (**C**) Association analyses between genotyped or best guess ($r^2 > 0.95$) *ERAP* SNP and typed HLA genotype (n=364), excluding all NLPHL cases. (**D**) Results of the *ERAP1* haplotypes (maximum probability > 0.9) and typed or best guess ($r^2 > 0.8$) HLA genotype association (n=374). A Chi-square test was used to determine significance of the associations. A p-value < 0.005 (dashed line) was considered significant.

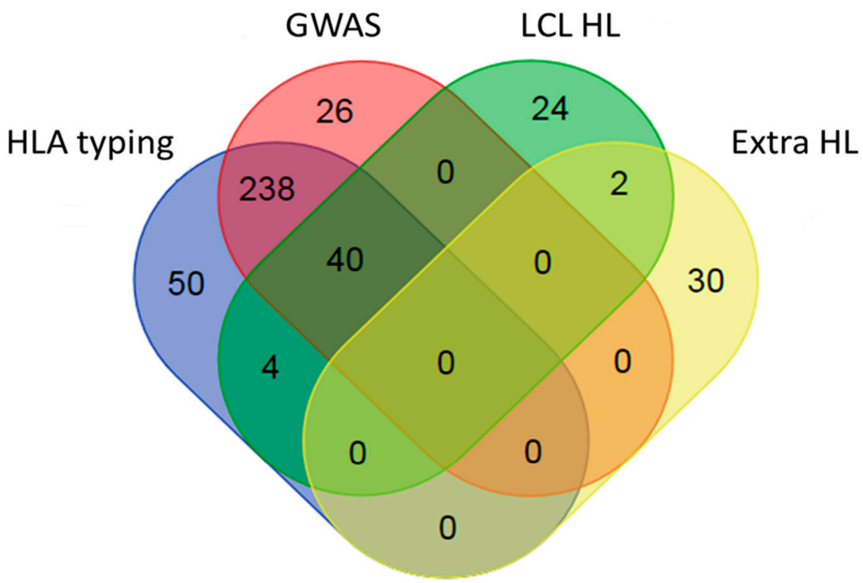

Figure S6. Schematic representation of the HL cases included in this study. Four partially overlapping HL patient groups were combined in this study: 1) 332 HL cases with available HLA type data from a previously published HLA typing study [6]. 2) 304 cases with SNP genotyping data from a previously published GWAS study [11]. 3) 70 HL cases from which we generated LCLs. 4) 32 extra HL cases with HLA typing data and genotyping for the selected *ERAP* SNPs.
